# Effect of a higher protein diet and lifestyle camp intervention on childhood obesity (The COPE study): results from a nonrandomized controlled trail with 52-weeks follow-up

**DOI:** 10.1007/s00394-024-03420-z

**Published:** 2024-05-09

**Authors:** Dorthe D. Jakobsen, Lea Brader, Jens M. Bruun

**Affiliations:** 1 Steno Diabetes Center Aarhus, Aarhus University Hospital, 8200 Aarhus N, Denmark; 2 Department of Clinical Medicine, Aarhus University, 8200 Aarhus N, Denmark; 3 Danish National Center for Obesity, 8200 Aarhus N, Denmark; 4 Arla Innovation Centre, Global Nutrition, 8200 Aarhus N, Denmark

**Keywords:** Childhood obesity, Diet, Protein, Lifestyle intervention, Children and adolescents, Overweight and obesity

## Abstract

**Purpose:**

In adults, diets rich in protein seem beneficial in relation to satiety, weight loss, and weight management; however, studies investigating dietary protein and weight development in children are scarce and inconsistent. This nonrandomized controlled trial aimed to investigate the effect of a higher protein diet during lifestyle intervention on anthropometry and metabolic biomarkers in children with overweight and obesity.

**Methods:**

Children (n:208) were recruited from two multicomponent lifestyle camps. One camp was assigned as the intervention group. In the intervention group, carbohydrates-rich foods at breakfast and two in-between-meals were replaced with protein-containing foods to increase the amount of protein from ~ 10–15 energy percent (E%) per day to ~ 25E% per day. Other components were similar between groups. Anthropometry and biochemical measurements were collected at baseline, 10 weeks (after camp) and 52 weeks.

**Results:**

The intervention group had a non-significant improvement in BMI-SDS (− 0.07 SD (− 0.19; 0.05), p = 0.24) compared to the control group, but in general, there was no effect of a higher protein diet on anthropometry and metabolic biomarkers. Overall, 10 weeks at camp resulted in a more favorable body composition [− 6.50 kg (p < 0.00), − 0.58 BMI-SDS (p < 0.00), and − 5.92% body fat (p < 0.00)], and improved metabolic health, with most changes maintained at 52 weeks.

**Conclusion:**

A higher protein diet had no significant effect on body composition and metabolic health; however, these lifestyle camps are an efficiatious treatment strategy for childhood obesity.

Clinical trial registration: clinicaltrials.gov with ID: NCT04522921. Preregistered August 21st 2020.

**Supplementary Information:**

The online version contains supplementary material available at 10.1007/s00394-024-03420-z.

## Introduction

The prevalece of obesity has rearched alarming proportions. The World Obesity Federation reports that 104 million children (5–9 years of age) and 151 million adolescents (10–19 years of age) will be living with obesity by 2030 [1, 2]. Danish trends for childhood obesity are comparable to those in other high-income countries. In 2021, 12–19% of Danish children (aged 6–15 years) had overweight and 3–4% obesity [3]. Overall, children with obesity have elevated cardiovascular risk factors and experience poorer health, lower self-esteem, and are subjected to stigma and bullying compared to peers with normal weight [3–6]. These conditions are very likely to persist into adulthood, with a higher risk of developing diseases such as cardioovascular diseases, cancers, and diabetes [4], underlining the importance of preventing and treating childhood obesity.

Currently, lifestyle interventions are the recommended treatment for children and adolescents with overweight and obesity [7, 8]. The majority of lifestyle interventions span for less than one year and focus only on diet and physical activity [9]. The most effective lifestyle interventions are multicomponent, including dietary modifications, increased physical activity, parental/family involvement, and behavioral therapy. Although, the quality of evidence is low [8, 10, 11], and beneficial effects are typically small and short-term [3, 12, 13]. An exercise-only or diet-only intervention is a more simplistic approach. Exercise-only interventions show some effect in reducing the risk of obesity in school-aged children and adolescents [14], but have not been superior to dietary advice alone in reducing visceral and subcutaneous adipose tissue and improving cardiometabolic biomarkers [5]. Most dietary interventions are implemented in schools [15]; however, current evidence shows no effect of diet-only interventions on childhood obesity outcomes [14, 16]. The only dietary advice proven by randomized controlled studies to reduce the risk of childhood obesity is a reduction in sugar-sweetened drinks [17–19], while the consumption of a diet high in dairy products may be beneficial in alleviating overweight and obesity in children and adolescents [20–24]. In adults, diets rich in protein seem beneficial in relation to satiety, weight loss, and weight management [25, 26]. In relation to weight loss, theoretically, a higher intake of protein increases energy expenditure through increased postprandial thermogenesis and resting metabolism, and further, contributes to higher satiety compared to the intake of carbohydrates and fats, possibly decreasing total energy intake [27]. However, in children, studies investigating the effect of increased dietary protein on weight development are scarce and results are conflicting [28–32]. Thus, currently no evidence is available to determine whether a specific macronutrient composition and a higher protein diet are favorable in managing childhood obesity [33].

The primary aim of the present study was to investigate whether a diet higher in protein-containing foods was more effective than the standard weight-loss diet served to children in the setting of a multicomponent lifestyle camp in achieving beneficial changes in anthropometry and metabolic biomarkers.

## Methods

**Fig. 1:**
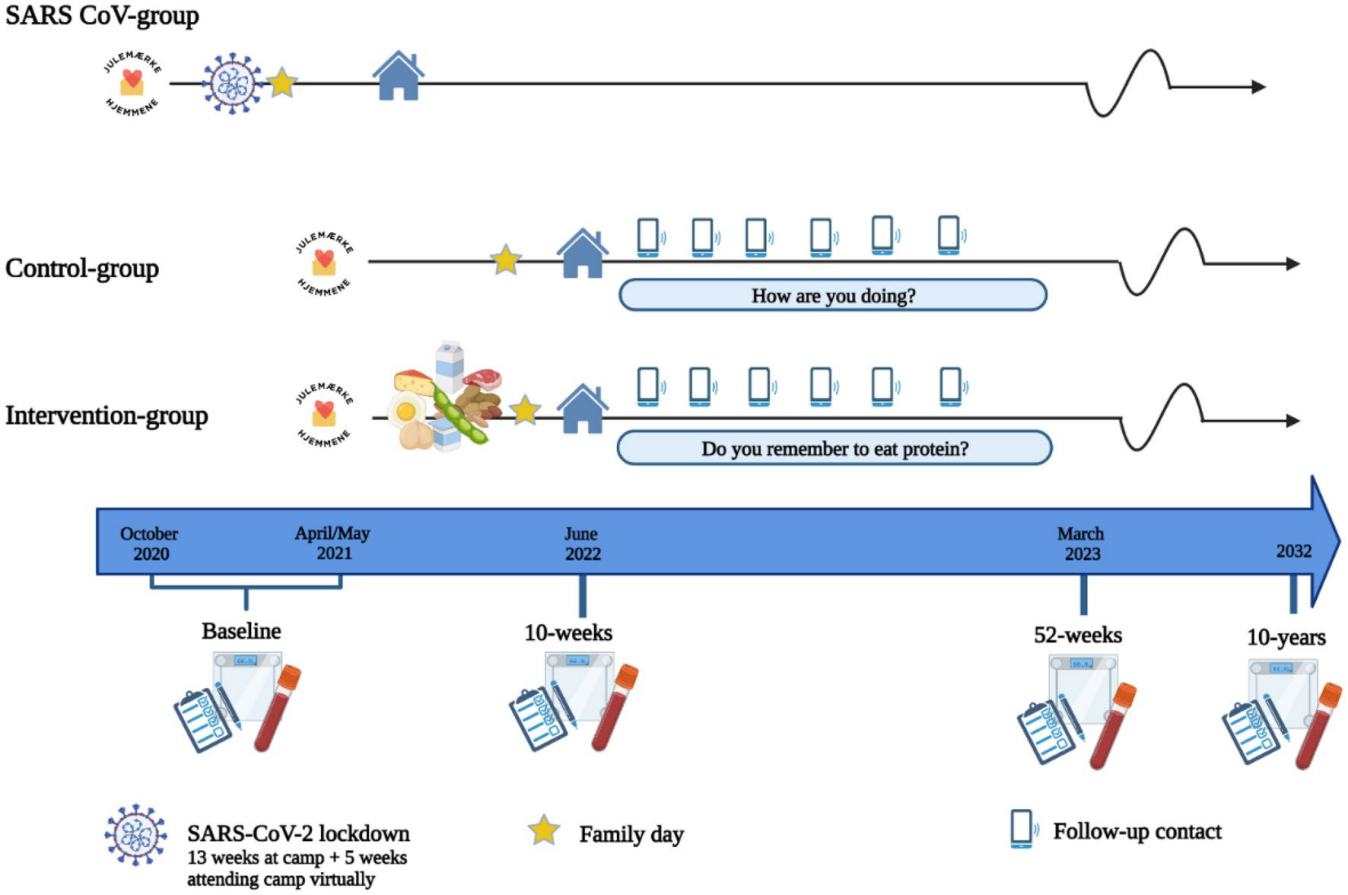
Study design of The COPE study. The figure is created with BioRender.com, and a publication license is granted

The Childhood Obesity—Prevention of Diabetes Through Changed Eating Patterns Study (referred to as The COPE study) is a prospective nonrandomized controlled trail conducted at multicomponent lifestyle camps in Denmark. The COPE study was designed to investigate the effect of a higher protein diet on weight loss and health-related outcomes in children from 7 to 14 years of age with overweight and obesity, and the study design is presented in Fig. 1.

The multicomponent lifestyle camps are managed by Julemærkefonden, a non-governmental organization that manage five multicomponent lifestyle camps in Denmark. Julemærkefonden and all five camps are financed by funding from investors and private companies, and children attend these camps free of charge. Children are referred to attend camp for 10 weeks by their general practitioner if they struggle with overweight or obesity, loneliness, unhappiness, or social or family-related problems. All camps are multicomponent, focusing on improving health and quality of life through social and physical activities, healthy meals, and daily exercise. At camp, children attend school for approximately three hours daily. During camp, social workers with experience in childcare support and motivate all children. Children go home to their families on weekends, and the families are invited to four family education days. After the 10-week camp, the children are supported to explore opportunities in their respective municipalities, which may include programs for obesity treatment and/or participation in sports activities. The child and their family revisit the camp for a concluding follow-up session, taking place approximately one month after their last day at camp. This provides an opportunity to engage in discussions with camp staff and peers, sharing their experiences and progress.

### Recruitment

Julemærkefonden is responsible for allocating children to the respective camps, usually placing them in the camp nearest to their homes. The study was initiated in collaboration with two of the camp sites (Julemærkehjem Hobro and Julemærkehjem Fjordmark). All children assigned camp Hobro from October 2020, and camp Fjordmark from May 2021 until March 2022 were invited to participate, and parents/guardians received information about the present study before starting camp. Camp staff invited children and parents/guardians to an individual information meeting four to eight weeks before starting camp, and if they agreed to participate in the study, parents/guardians were asked to sign a written consent for their child to participate, and one parent/guardian were asked to sign a written consent for themselves to participate with their child. Children were excluded from the study if they were diagnosed with an eating disorder or a disease requiring a special diet. Children or parent/guardians who participated in another clinical trial or did not understand or were unwilling/unable to comply with the study protocol were also excluded.

### Intervention

All camps are managed according to the same rules and regulations and comply to the official Danish guidelines concerning diets and physical activity [34, 35]. According to camp dietary policy, children are served six meals daily, and all meals are provided with instructions on portion sizes. The daily energy intake ranges from 1200 to 1800 kilocalories depending on age. For example, children below 10 years of age may be served less rye bread, potatoes, or rice. At breakfast, children are allowed to drink two cups of milk and eat three half slices of rye bread with various toppings and greens/fruit. One half slice of rye bread can be substituted with a bowl of cereal. At lunch, children are served a warm prepared meal with one piece of meat, salad/vegetables/legumes, and potatoes, rice, or pasta. At dinner, children younger than 10 years of age are allowed to eat three half slices of rye bread, while children above 10 years of age are allowed to eat four half slices of rye bread with different toppings and greens/vegetables. Additionally, children are served three in-between meals: half a piece of fruit before lunch, and one crispbread/bun with high fiber content plus greens/fruit as an afternoon and evening snack. Water is served ad libitum. According to the camp dietary policy, the targeted distribution of macronutrients is 10–15 energy percent (E%) protein per day, 45–60E% carbohydrate per day, and 25–40E% fat per day in accordance with official Danish dietary guidelines [34]. Additionally, certain foods, such as candy, sweets, and soda, are restricted.

Due to ethical considerations and the daily routines at camp (Supplementary S1), it was not feasible to randomize children within camps. Both camps comply with the same dietary policy, rules, and routines concerning meals and physical activity. Since camp Hobro have the capacity to recruit 250 children per year compared to 150 children per year at camp Fjordmark, camp Fjordmark was assigned as the active control group, and camp Hobro was assigned as the intervention group. In the control group, no changes were made. In the intervention group, the aim was to replace carbohydrates-rich foods at breakfast and two in-between-meals with naturally protein-containing foods (e.g., dairy products, nuts, egg, meat-products) to increase the amount of protein from ~ 10–15E% to ~ 25E% per day with minimal changes in total caloric intake. Meal changes were planned in collaboration with the kitchen staff to increase compliance, and the kitchen staff were provided with a weekly meal schedule and instructions on how to alternate between different protein-containing foods to ensure variety and accommodate special needs. Kitchen and camp staff in the intervention group motivated all children to eat the protein-containing foods. No changes in fruit/vegetables/salads were made, and kitchen staff were instructed to serve fruit/vegetables/salads as usual.

Halfway through the camp, children and parents/guardians in both groups were invited to a family education day. Both groups participated in social and physical activities and were introduced to the official Danish dietary guidelines [36]. Furthermore, the intervention group had a 30-min educational class focusing on protein in relation to weight loss, satiety, and physical activity. They learned how to identify protein-containing foods well-known from the supermarket, received a pamphlet containing suggestions for protein-rich breakfast and in-between meals, and were encouraged to continue eating a higher protein diet after camp. The remaining family education days were carried out by camp staff as usual.

Once a month for the first six months after camp, study staff contacted all families through text messages to ask if they would like a follow-up phone call. The aim was to motivate the intervention group to continuously consume a higher protein diet and support health-promoting behaviors in the control group. On average, study staff were in contact with the families two times within that period, and 22% of the families never received follow-up phone calls; therefore, no further analyses of this intervention strategy were performed.

### Measurements

Body weight (kg), body fat (%), and skeletal muscle mass (kg) were measured in light clothing without shoes using a Bioelectric impedance (InBody model 270, Hopkins Medical Products, Grand Rapids, MI, USA). Height (m) was measured using a fixed wall measuring tape. BMI-SDS was calculated using World Health Organization AnthroPlus software and considered the primary outcome. A BMI-SDS > 1SD was defined as overweight, and a BMI-SDS > 2SD was defined as obesity [37]. Furthermore, blood pressure was measured with the right arm placed at heart level using an automatic non-invasive blood pressure monitor (Omron M3, Kyoto, Japan). Camp staff were responsible for measuring anthropometry and blood pressure in all children at baseline, 10 and 52 weeks.

Blood samples were optional for all children. Educated bio-analysts at Aarhus University Hospital were responsible for collecting blood samples from a subsample of the children at baseline, 10 and 52 weeks.

### Questionnaires

Children and parents/guardians answered several questionnaires, and all questionnaires were delivered electronically to the participating parent/guardian using REDcap.org database located at Aarhus University.

Background characteristics were collected with a parent-reported questionnaire, including child sex and age, parental education, household income, diseases in the family, participation in physical activity, etc. In accordance with national physical activity guidelines [38], the authors formulated three questions to evaluate physical activity behavior among the children, specifically focusing on high intensity, moderate intensity, and the settings of physical activities. Quality of life was measured using the validated Danish version of the Pediatric Quality of Life Inventory 4.0 questionnaire (PedsQL 4.0) [39]. The Children’s Eating Habits Questionnaire-FFQ (CEHQ-FFQ) [40] was translated into Danish by the authors, and a few foods were added to investigate eating habits. Eating behavior was measured using a Danish version of the Child Eating Behavior Questionnaire (CEBQ) [41]. The prevalence of subjective binge eating and loss-of-control eating was measured using two questions from the Eating Disorder examination questionnaire (EDE-Q 6.0) [42, 43].

Child and parents/guardians answered background characteristics at baseline. Physical activity behavior among children was assessed at baseline and 52 weeks, but not at 10 weeks, as all children were engaged in physical activity in line with national recommendations during camp. All other questionnaires were answered at baseline, 10 and 52 weeks.

Separate papers will be published presenting changes in quality of life, physical activity behavior, eating habits, eating behavior and binge eating/loss-of-control eating.

### Statistics

As illustrated in Fig. 1, the COVID-19 pandemic forced a lockdown from December 2020 to February 2021. All children affected by the COVID-19 lockdown were sent home for five weeks with no control of dietary intake and physical activity, which is why they were excluded. Furthermore, due to the COVID-19 lockdown, meal changes in the intervention group were postponed from April to May 2021, and few children starting camp in March/April 2021 were therefore served a standard protein-diet for 4–6 weeks and a higher-protein diet for 4–6 weeks. These children were excluded whenever investigating differences in changes between groups.

The power calculation was based on a previous study investigating the effect of a higher protein intake (21E% vs. 32E%) on childhood obesity [30]. In this study, 22 and 24 children were allocated to the standard and high protein diet, respectively. Both groups had a reduction in BMI z-score during the 13-week intervention (from 2.48 ± 0.06 BMI-z to 2.10 ± 0.10 BMI-Z in the high protein group, and from 2.51 ± 0.05 BMI-Z to 2.40 ± 0.10 BMI-Z in the standard protein group), with a greater reduction in the high protein group (p = 0.03) [30]. The number of participants needed to detect a between-group difference of − 0.27 SD with a power of 80% and a significance level of 5% was calculated to be ~ 55 children in each group.

Continuous data are presented as mean ± standard deviation (SD) for parametric data and median [inter quartile range (IQR)] for non-parametric data. Categorical data are presented as absolute numbers and percentage (%). Differences between groups at baseline were tested using linear regression analysis. As this study investigate a rather homogenous group, e.g., with a lower socioeconomic position compared to children with overweight and obesity not attending camp [44], we assume that missing data are randomly distributed across variables. Therefore, unadjusted mixed effects models were performed to investigate differences in change between the groups. All mixed effect models accounted for repeated measures over time at the individual level, thus controlling for differences in children’s weight based on their own baseline measurements. Group level was not included as a random effect, but as an interaction term to evaluate potential variations in the intervention effect across groups.

QQ-plot of the residuals was used to check for normal distribution errors. Linearity and identical distribution errors were assessed by checking the residual plots versus the predicted values. If necessary, log-transformation of the data was performed to reduce the skewness of data to meet the assumptions of linearity and equal distribution errors. Sensitivity analyses including only participants with complete data were performed to investigate the robustness of the primary outcome (BMI-SDS). Additionally, intention-to-treat analyses were performed on the complete sample, potentially reflecting the real effect of the camps more accurately.

All statistical analyses were performed using Stata/MP 17.0 (StataCorp LLC, USA) with a p < 0.05 considered statistical significant.

## Results

**Fig. 2:**
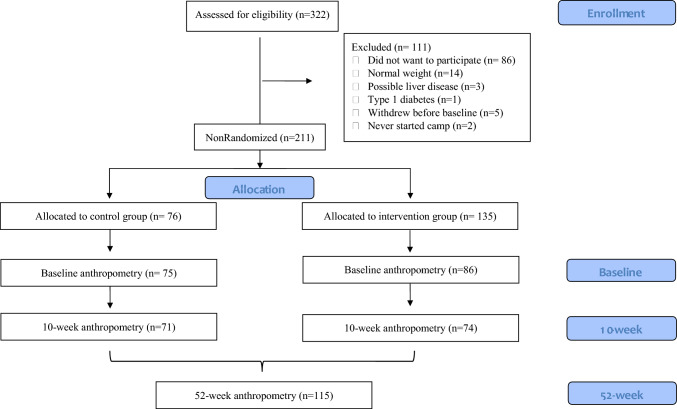
Flowchart of study participants

In the COPE study, 322 children were invited to participate, and 236 accepted the invitation (~ 73% response rate). Children were excluded for various reasons, e.g., if they were identified with possible liver disease (alanin aminotransferase (ALAT) > 80) or if they had a normal weight according to WHO definitions. After allocation, one child from the control group and two children from the intervention group dropped out of camp. Furthermore, from the intervention group, 34 children were excluded due to the COVID-19 lockdown, and 13 children were excluded as they were served a higher-protein diet for less than six weeks. At baseline, 75 children were included in the control group and 86 children in the intervention group, with a subsample of those children having a blood sample collected (Fig. 2). Children in the intervention group had higher total cholesterol and higher low density lipoprotein (LDL) cholesterol compared to children in the control group, but otherwise, the groups did not differ in anthropometry and metabolic biomarkers at baseline (Table 1). Overall, children had a mean age of 12.2 ± 1.4 years and a BMI-SDS of 2.7 ± 0.7 SD, with 15% defined as having overweight and 85% defined as having obesity according to the WHO Child Growth Standards [37].

**Table 1:** Baseline characteristics (n:161)

	All (n:161)	Control group (n = 75)	Intervention group (n = 86)	Differences between groups at baseline
Age (years)	12.2 ± 1.4	12.4 ± 1.4	12.1 ± 1.5	p = 0.12
Sex				
Male	71 (44%)	36 (48%)	35 (41%)	p = 0.35
Female	90 (56%)	39 (52%)	51 (59%)	
Weight (kg)	73.2 ± 16.5	73.6 ± 14.6	72.7 ± 18.0	p = 0.73
Height (cm)	1.59 ± 0.1	1.59 ± 0.1	1.59 ± 0.1	p = 0.67
BMI-SDS (WHO)	2.65 ± 0.7	2.65 ± 0.7	2.66 ± 0.7	p = 0.93
Weight class (WHO)				
OW	24 (15%)	13 (17%)	11 (13%)	p = 0.42
OB	137 (85%)	62 (83%)	75 (87%)	
Body fat (%)	41.2 ± 6.7, n:151	41.4 ± 6.6, n:67	41.0 ± 6.8, n:84	p = 0.67
Skeletal muscle mass (kg)	23.1 ± 5.4, n:151	23.0 ± 4.8, n:67	23.2 ± 5.8, n:84	p = 0.80
Systolic BP (mmHg)	110.5 ± 10.5, n:141	111.1 ± 10.7, n:73	109.8 ± 10.31, n:68	p = 0.45
Diastolic BP (mmHg)	71.3 ± 7.7, n:141	70.2 ± 6.4, n:73	72.5 ± 8.7, n:68	p = 0.07

	All (n:75)	Control group (n = 33)	Intervention group (n = 44)	
P-Cholesterol (mmol/L)	4.2 ± 0.7	3.9 ± 0.7	4.3 ± 0.7	p = 0.02
HDL Cholesterol (mmol/L)	1.2 ± 0.2	1.2 ± 0.3	1.2 ± 0.2	p = 0.24
LDL Cholesterol (mmol/L)	2.4 ± 0.6, n:73	2.2 ± 0.6	2.5 ± 0.6, n:40	p = 0.01
P-Triglyceride (mmol/L)	1.3 (0.9;1.6)	1.3 (0.9;1.5)	1.3 (0.9;1.8)	p = 0.42
ALAT (units/L)	22 (18;30)	22 (18;27)	22.5 (18;33)	p = 0.36
ASAT (units/L)	23 (20;27)	24 (20;25)	23 (20;29)	p = 0.29
GGT (units/L)	16 (13;19)	16 (14;19)	16 (13;19)	p = 0.58
Basic phosphatase (units/L)	284 ± 87	297 ± 84	274 ± 90	p = 0.12
HbA1c (mmol/mol)	34.9 ± 2.3	34.9 ± 2.5	35.0 ± 2.1	p = 0.87
P-glucose (mmol/L)	5.9 ± 0.3	5.9 ± 0.4	5.9 ± 0.3	p = 0.99
Albumin (g/L)	42.4 ± 2.4	42.0 ± 2.4	42.8 ± 2.4	p = 0.14
CD 163 (mg/L)	2.5 (2;3.1)	2.4 (2;3.1)	2.6 (2.1;3)	p = 0.78
Platelets (units/L)	312 ± 62	314 ± 64	310 ± 61	p = 0.80
Uric acid (mmol/L)	0.31 ± 0.1	0.31 ± 0.0	0.32 ± 0.1	p = 0.54

Data are presented as mean ± standard deviation (SD) for parametric data and median (inter quartile range (IQR)) for non-parametric data. Linear regression model used to test for differences between groups at baseline. Overweight (OW) = BMI−SDS > 1SD. Obesity (OB) = BMI−SDS > 2SD

### Dietary intervention

Based on three different combinations of breakfast, afternoon, and evening snacks, the macronutrient composition in the control group were 71E% carbohydrate, 19E% protein, and 11E% fat per day, equivalent to 438 kcal. In comparison, the macronutrient composition served for children in the intervention group were 42E% carbohydrate, 33E% protein, and 24E% fat per day, equivalent to 417 kcal (Supplementary S2). Total caloric intake and leftovers were not measured in either group, and remaining meals (before-noon snack, lunch, and dinner) were not measured but served in accordance with camp dietary policy in both groups.

### Effect of a higher protein diet on anthropometry and metabolic biomarkers

From baseline to 10 weeks, there were no differences between groups in anthropometry or metabolic biomarkers. Although non-significant, children in the intervention group tended to have a greater decrease in BMI-SDS, but with no difference between groups in body fat and skeletal muscle mass. No significant differences were found between groups in metabolic biomarkers; however, children in the intervention group tended to have a greater decrease in LDL cholesterol, ALAT, aspartate transaminase (ASAT), and platelets (Table 2). Analyses on complete data did not change the result of the primary outcome (BMI-SDS).

**Table 2:** Differences in change between groups from baseline to 10 weeks

	Δ (95% CI)	p-value
Weight (kg)	− 0.39 (− 2.19, 1.40)	p = 0.67
Height (cm)	0.00 (− 0.00, 0.01)	p = 0.15
BMI-SDS (WHO)	− 0.07 (− 0.19, 0.05)	p = 0.24
Body fat %	− 0.28 (− 1.87, 1.30)	p = 0.73
Skeletal muscle mass (kg)	− 0.22 (− 0.79, 0.35)	p = 0.45
Systolic BP (mmHg)	1.06 (− 2.42, 4.55)	p = 0.55
Diastolic BP (mmHg)	− 1.31 (− 3.88, 1.27)	p = 0.32
P-Cholesterol (mmol/L)	− 0.17 (− 0.45, 0.11)	p = 0.23
HDL Cholesterol (mmol/L)	− 0.02 (− 0.11, 0.07)	p = 0.65
LDL Cholesterol (mmol/L)	− 0.19 (− 0.43, 0.05)	p = 0.13
P-Triglyceride (mmol/L)	− 0.03 (− 0.27, 0.21)^a^	p = 0.83
ALAT (units/L)	− 0.14 (− 0.33, 0.05)^a^	p = 0.15
ASAT (units/L)	− 0.12 (− 0.28, 0.04)^a^	p = 0.13
GGT (units/L)	− 0.06 (− 0.23, 0.10)^a^	p = 0.45
Basic phosphatase (units/L)	11.73 (− 18.34, 41.81)	p = 0.44
HbA1c (mmol/mol)	0.26 (− 0.72, 1.23)	p = 0.61
p-glucose (mmol/L)	0.05 (− 0.10, 0.19)	p = 0.52
Albumin (g/L)	0.37 (− 0.75, 1.49)	p = 0.52
CD 163 (mg/L)	− 0.08 (− 0.21, 0.04)^a^	p = 0.20
Platelets (units/L)	0.04 (− 0.02, 0.10)^a^	p = 0.16
Uric acid (mmol/L)	0.01 (− 0.01, 0.03)	p = 0.48

Mixed effect models used to investigate differences in change from baseline to 10 weeks (after camp) between control group (ref.) and intervention group. No adjustments

^a^Analyses performed on log-transformed data

### Effect of lifestyle camp intervention on anthropometry and metabolic biomarkers

Due to no difference in change between the control group and intervention group, the groups were pooled to investigate the overall effect of the 10-week lifestyle camp on anthropometry and metabolic biomarkers. For this purpose, data were included from 191 children (88 with blood samples) at baseline, from 171 children (73 with blood samples) at 10 weeks, and from 115 children (43 with blood sample) at 52 weeks.

**Fig. 3:**
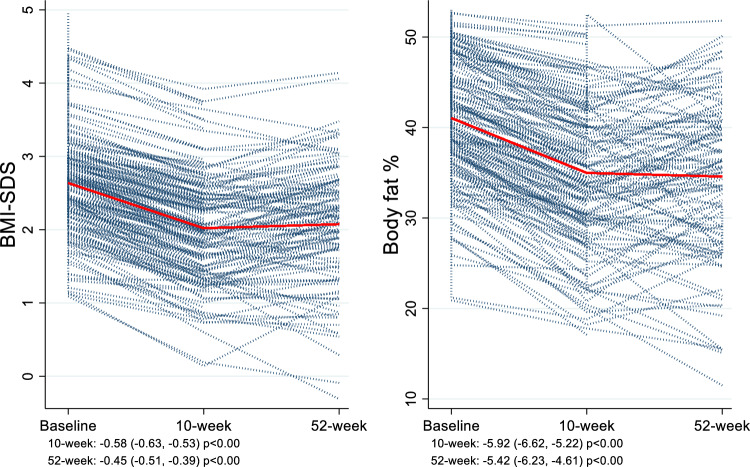
Line plots showing change from baseline to 10 and 52-weeks in BMI-SDS and body fat. Blue lines showing individual changes. Red lines showing mean changes

From baseline to 10 weeks, anthropometry improved for all children. On average, children reduced their body weight by 6.50 kg [(− 7.30; − 5.69), p < 0.00], BMI-SDS by 0.58 SD, and body fat by 5.92%, with no changes in skeletal muscle mass [(0.06 kg (− 0.20; 0.32), p = 0.64] (Fig. 3).

From 10 to 52 weeks, one child was excluded due to a significant increase in liver markers (i.e., ALAT, ASAT, and gamma-glutamyltransferase (GGT)) suggestive of inflammatory liver disease.

At the 52-week follow-up, 86% of children continued to have a lower BMI-SDS compared to baseline. On average, BMI-SDS and body fat were lower at 52 weeks compared to baseline, with an expected increase in body weight (1.77 kg (0.83; 2.71), p < 0.00) and skeletal muscle mass (2.85 kg (2.55; 3.15), p < 0.00) due to growth (Fig. 3).

Systolic (− 3.86 mmHg (-5.45; − 2.27) p < 0.00) and diastolic blood pressure (− 3.83 mmHg (− 5.04; − 2.62), p < 0.00) were reduced after 10 weeks. At 52 weeks, the diastolic blood pressure was still reduced compared to baseline (− 2.75 mmHg (− 4.10; − 1.40) p < 0.00) (Supplementary S3).

**Fig. 4:**
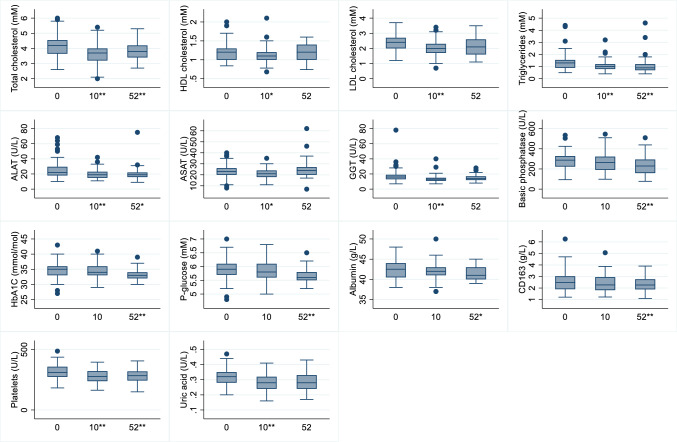
Box plots of change in metabolic biomarkers from baseline to 10 and 52-weeks. Boxes represents the IQR with the blue line showing the median. Whiskers represent highest and lowest values with outliers. **p < 0.00, *p < 0.05

On average, children had reduced levels of total cholesterol, high density lipoprotein (HDL) cholesterol, LDL cholesterol, triglycerides, ALAT, ASAT, GGT, platelets, and uric acid after 10 weeks. At 52 weeks, total cholesterol, triglycerides, ALAT, basic phosphatase, HbA1c, P-glucose, albumin, CD163, and platelets were reduced compared to baseline (Fig. 4).

Analyses on complete data did not change the result of the primary outcome (BMI-SDS); however, dropout analysis showed that children missing at 52 weeks had a higher BMI-SDS (p < 0.00) at baseline compared to children participating in the 52-weeks follow-up. The results of the intention-to-treat analyses were consistent with the per-protocol analyses, as detailed in Supplementary S4.

Additional information regarding the changes in physical and metabolic health from baseline to 10 and 52 weeks is available in Supplementary S3.

## Discussion

Based on the present study, there were no additional effect of consuming more protein-containing foods during a multicomponent lifestyle intervention on anthropometry and metabolic biomarkers in children with overweight and obesity. However, children in the intervention group showed a numerical but non-significant improvement in body composition compared to children in the control group. Based on the intervention meals (i.e., breakfast, afternoon, and evening snacks), the differences in protein between groups were on average 14E% of protein per day. According to kitchen and camp staff, most children in the intervention group consumed the protein-containing foods; however, both groups may have had leftovers, which were not measured, and based on studies in adults, dietary compliance is the primary reason for discrepant findings of the effect of protein [27]. Additionally, it was not possible to control or estimate lunch, dinner, and fruit/vegetables served with in-between meals in either group. Furthermore, physical activities arranged by camp staff, which may have varied in intensity and duration between groups, could have affected the absolute difference in protein and total energy expenditure. Moreover, children participating in the present study were healthy children with overweight or obesity, and a meta-analyses suggest that adults with prediabetes benefit more from a diet high in protein [25].

Only one previous study has reported a high-protein low-carbohydrate diet (32E% protein per day) to be an effective and safe strategy to reduce BMI z-score in children with severe obesity compared to a low-fat diet (21E% protein per day) [30]. Two more recent studies reported no difference in BMI z-score of a higher protein diet [29, 31]. Nevertheless, in these two studies, the difference in protein between groups was only 4-5E%, which may explain these discrepancies. A meta-analysis investigating the effects of different macronutrient compositions in children found no effect on BMI z-score, body fat, or lean body mass, but a small tendency favoring a higher protein diet compared to a higher carbohydrate diet [24], underlining that it might be challenging to investigate the effect of different macronutrient compositions. This is because an increase in one macronutrient, e.g., protein, will force other macronutrients to change, making it difficult to determine causality. Furthermore, when investigating the effect of macronutrient composition on weight development, it is important to consider and specify the food sources because different food sources may have an effect per se and provide different types of e.g., protein, other nutrients, and bioactive components [24, 45].

Since no differences were observed between the control group and intervention group, the authors chose to investigate the overall effect of these unique multicomponent lifestyle camps on anthropometry and metabolic biomarkers. Based on the present results, 10 weeks at camp were highly effective in reducing body weight, BMI-SDS, and body fat while retaining muscle mass. From 10 weeks to the 52-week follow-up, children maintained a lower BMI-SDS and body fat compared to baseline despite an increase in body weight, which was expected due to growth. Furthermore, children had a reduction in cardiometabolic risk factors, liver markers, and uric acid after 10 weeks, suggesting favorable changes in metabolic health, and many of these changes were sustained at the 52-week follow-up.

The effectiveness of these unique multicomponent lifestyle camps has previously been investigated in 2012. In contrast to the 2012 study, the present study found a more robust and sustained reduction in BMI-SDS after 52 weeks, with only a 0.05 SD increase compared to an increase of 0.19 SD in the 2012 study. This is of interest since children in the 2012 study had a slightly higher BMI-SDS (2.93 SD), and similar BMI-SDS reductions were observed after the 10-week intervention [46]. In addition, favorable biochemical changes achieved at 10 weeks were to a greater extent maintained at 52 weeks in the present study. However, dropout analyses also showed a higher BMI-SDS at baseline among children who did not complete the 52-week follow-up, inducing a potential risk of selection bias. Over the past decade, these lifestyle camps have changed from being solely weight loss camps to also providing support for children who are lonely, unhappy, or facing social or family-related problems. Additionally, these camps have increased their focus on collaborating with parents/guardians and the municipalities compared to before, which could explain why the present cohort showed greater maintenance of BMI-SDS and sustained beneficial biochemical health changes after 52 weeks.

In general, lifestyle interventions are a preferable and safe treatment option, proven to be more effective in achieving weight loss compared to no intervention or usual care [8, 17, 47]. However, the degree of weight loss is only moderate, and lifestyle interventions seem most effective in younger children with overweight, emphasizing the importance of an early intervention strategy [17]. Furthermore, evidence shows that lifestyle interventions are most effective when they include parental involvement, group interaction, and last for more than six months [8, 10, 11, 17]. In the present study, children were moved from their home environment without parents/guardians, placed in a group of peers, and usually followed for only two months after camp, which is a unique setting not seen elsewhere and partly contradictory to current recommendations. Still, the present study clearly demonstrated that most children improved their physical and metabolic health during camp and that these beneficial health changes were maintained one year later, suggesting that these camps provide a beneficial environment for behavioral change. In accordance with the study protocol, the authors plan to investigate changes after 3, 5, 7, and 10 years to explore if this unique short-term intervention can prevent children with overweight and obesity from developing obesity and concomitant diseases in adulthood.

## Limitations

The present study has some limitations that should be considered when interpreting the results. First, girls entering puberty naturally undergo changes in fat mass, so any potential increase in fat mass could be attributed to puberty status rather than solely to the intervention. However, we believe that changes in puberty status over the 10-week period were minimal and thus did not affect the estimated group differences. Second, total caloric intake was not measured in either group, and leftovers were not accounted for, potentially making the protein gap between groups smaller than actually reported. Third, although all anthropometric measurements were conducted in accordance with general recommendations, they were performed by different camp staff members, which could introduce measurement bias. Fourth, physical activity was assessed by questionnaire at baseline and 52 weeks, so adjustments for energy expenditure was not possible. However, during the camp, all children engaged in physical activity consistent with Danish recommendations, likely minimizing the impact on estimates from baseline to 10 weeks. Finally, from 10 to 52 weeks, children may have participated in different interventions depending on opportunities within their municipality. Therefore, the observed effects at 52 weeks could also be influenced by unknown interventions during that period.

## Conclusion

The present study found no favorable additive effect of a higher protein diet compared to a standard weight-loss diet following official dietary guidelines in a multicomponent lifestyle setting, although a numerical but non-significant improvement in body composition was observed. Overall, children attending these lifestyle camps achieved favorable changes in anthropometry and metabolic biomarkers after 10 weeks at camp. These changes, observed in both anthropometry and most biochemical markers, were maintained at the one-year follow-up. Thus, this short-term multicomponent lifestyle intervention appears to be a highly effective and safe treatment option for children with overweight and obesity. However, further research is needed to investigate the psychosocial and long-term effects.

## Supplementary Information

Below is the link to the electronic supplementary material.

Supplementary file 1 (DOCX 680 KB)

## Data Availability

The data used and/or analyzed in the present study is available from the corresponding author on reasonable request.

## Declarations

### Reporting checklist

The authors have completed the CONSORT reporting checklist.
