# Supplementary material for: Effect of a higher protein diet and lifestyle camp intervention on childhood obesity (The COPE study): results from a nonrandomized controlled trail with 52-weeks follow-up
Source: Eur J Nutr. 2024 May 9;63(6):2173–84. doi: 10.1007/s00394-024-03420-z (PMC11377484; doi:10.1007/s00394-024-03420-z)
Supplement: Supplementary file 1 — (DOCX 680 KB) [file 394_2024_3420_MOESM1_ESM.docx]

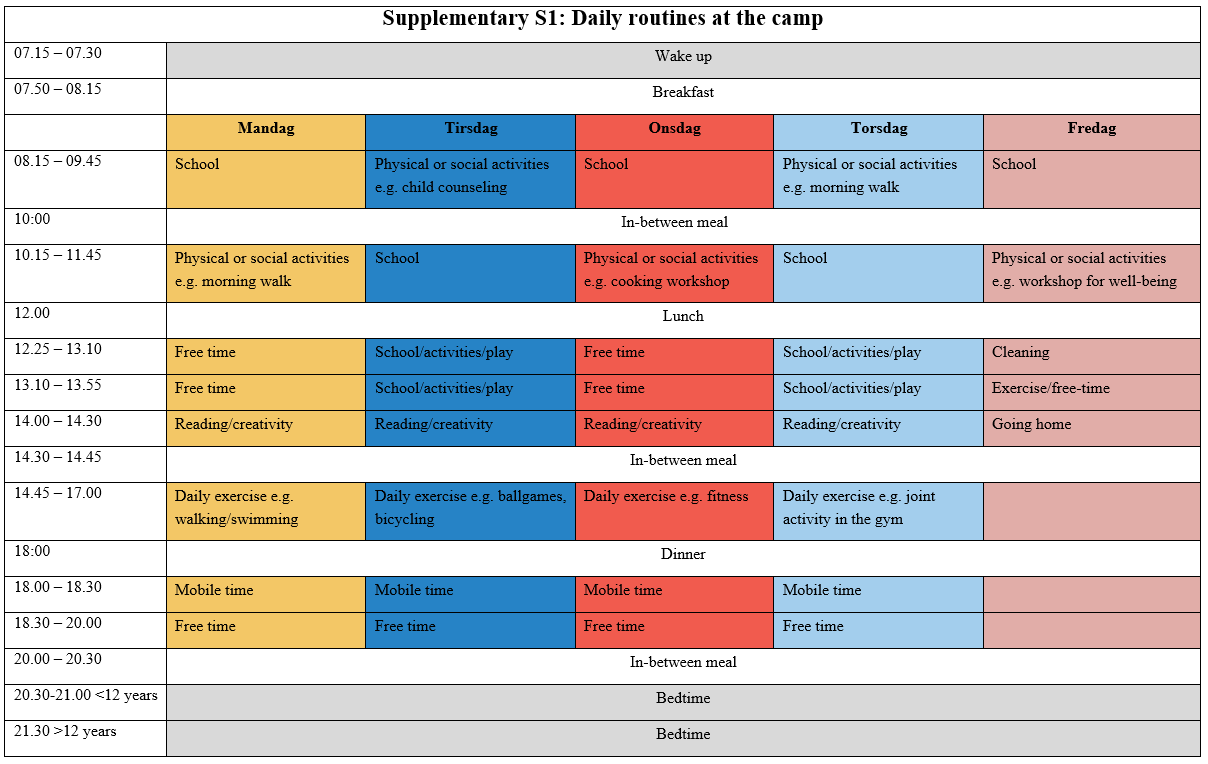


| **Supplementary S2: Dietary intervention meals** | | |
| --- | --- | --- |
|  | Control group* | Intervention group* |
| Breakfast | 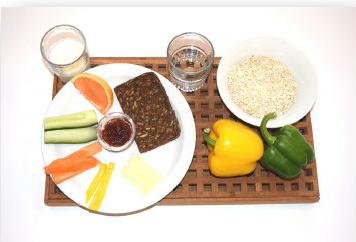*3 half slices of rye bread and 2 cups of milk* ***OR*** *2 half slices of rye bread with 30 g. of oat/rye cereal and 2 cups of milk*  *+ toppings, greens/fruit* | *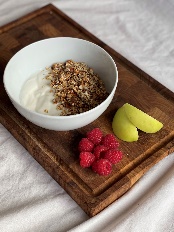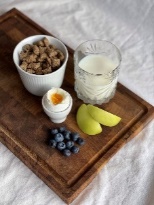*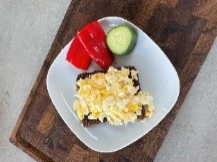  *2 half slices of rye bread with 2 eggs* ***OR*** *30 g. of oat/rye cereal with 1 ½ cups of milk and 1 boiled egg* ***OR*** *2 cups of high protein yoghurt with toppings*  *+ greens/fruit* |
| Before noon-snack | According to camp dietary policy – no changes | According to camp dietary policy – no changes |
| Lunch | According to camp dietary policy – no changes | According to camp dietary policy – no changes |
| Afternoon snack (2:30 PM) | 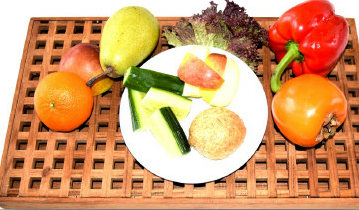  *1 crisp bread/bun with high fiber content*  *+ greens/fruit* | *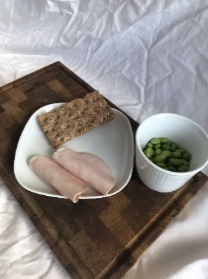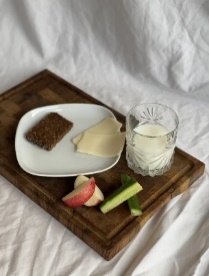*  *1 crisp bread* ***OR*** *½ half slice of rye bread with 30-40g low fat meat* ***OR*** *20 g. cheese*  *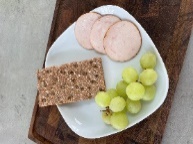+ greens/fruit* |
| Dinner | According to camp dietary policy – no changes | According to camp dietary policy – no changes |
| Evening snack (8:30 PM) | 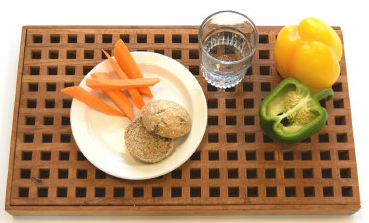  *1 crisp bread/bun with high fiber content*  *+ greens/fruit* | *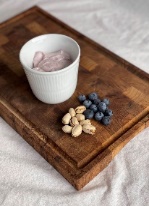*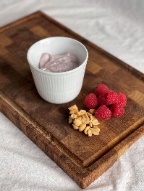  *1 cup of high protein yoghurt with 1 tablespoon of almonds* ***OR*** *peanuts* ***OR*** *pistachios*  *+ greens/fruit*  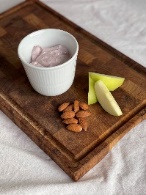dfbher |
| Average based on the three meals displayed. Greens/fruits not included. | 438 kcal (405-475 kcal) 71 E% carbohydrate (68-75 E% carbohydrate)  19 E% protein (16-20 E% protein)  11 E% fat (8-12 E%. fat) | 417 kcal (410 – 421 kcal) 42 E% carbohydrate (34-47 E% carbohydrate)  33 E% protein (30-36 E% protein)  24 E% fat (15-34 E% fat) |
| **All meals are served with fruit/vegetables of own choice – no changes made in intervention group.* | | |

| Supplementary S3: Changes in physical and metabolic health from baseline to 10 weeks and 52 weeks  (control group and intervention group pooled) | | | | | |
| --- | --- | --- | --- | --- | --- |
|  | **Baseline (n:191)** | **10 weeks (n:171)** | **52 weeks (n:115)** | **Changes from baseline to 10 weeks** | **Changes from baseline to 52 weeks** |
| Sex   - Male - Female | 85 (45%)  106 (55%) | 75 (44%)  96 (56%) | 50 (43%)  65 (57%) | p=0.81 | |
| Weight (kg) | 73.3±15.9 | 66.1±13.7 | 73.7±14.2 | -6.50 (-7.30, -5.69) p<0.001 | 1.77 (0.83, 2.71) p<0.001 |
| Height (cm) | 1.59±0.1 | 1.61±0.1 | 1.65±0.1 | 0.01 (0.01, 0.02) p<0.001 | 0.06 (0.05, 0.06) p<0.001 |
| BMI-SDS (WHO) | 2.6±0.7 | 2.0±0.7 | 2.1±0.8 | -0.58 (-0.63, -0.53) p<0.001 | -0.45 (-0.51, -0.39) p<0.001 |
| Weight class (WHO)   - NV (excluded at baseline) - OW - OB | 0 (0%)  30 (16%)  161 (84%) | 11 (6%)  75 (44%)  85 (50%) | 12 (10%)  38 (33%)  65 (57%) | p<0.01 | |
| Body fat % | 41.0±6.8, n:181 | 35.0±7.7, n:169 | 34.6±8.6, n:114 | -5.92 (-6.62, -5.22) p<0.001 | -5.42 (-6.23, -4.61) p<0.001 |
| Skeletal muscle mass (kg) | 23.3±5.2, n:181 | 23.3±5.3, n:169 | 26.2±5.4, n:114 | 0.06 (-0.20, 0.32) p=0.64 | 2.85 (2.55, 3.15) p<0.001 |
| Systolic BP | 110.4±10.6, n:162 | 106.6±11.1, n:156 | 111.1±11.0 | -3.86(-5.45, -2.27) p<0.001 | 0.84 (-0.95, 2.62) p=0.36 |
| Diastolic BP | 71.3±7.5, n:162 | 67.3±7.2, n:156 | 67.8±8.2 | -3.83 (-5.04, -2.62) p<0.001 | -2.75 (-4.10, -1.40) p<0.001 |
|  | **n:88** | **n:73** | **n:43** |  |  |
| P-Cholesterol | 4.2±0.7 | 3.7±0.7 | 3.9±0.7 | -0.52 (-0.65, -0.39) p<0.001 | -0.22 (-0.37, -0.07) p=0.004 |
| HDL Cholesterol | 1.19±0.2 | 1.15±0.2 | 1.20±0.2 | -0.06 (-0.10, -0.01) p=0.008 | -0.01 (-0.06, 0.04) p=0.695 |
| LDL Cholesterol | 2.36±0.6, n:86 | 2.02±0.6 | 2.17±0.6, n:42 | -0.37 (-0.48, -0.25) p<0.001 | -0.12 (-0.26, 0.01) p=0.07 |
| P-Triglyceride | 1.3 (0.9;1.6) | 1.0 (0.8;1.2) | 0.9 (0.7;1.2) | -0.19 (-0.29, -0.08) p=0.001† | -0.21 (-0.34, -0.08) p=0.002† |
| ALAT | 22 (18;29.5) | 19 (15;23) | 19 (16;22) | -0.17 (-0.25, -0.09) p<0.001† | -0.15 (-0.25, -0.04) p=0.005† |
| ASAT | 23 (20;26) | 21 (18;24) | 24 (20;27) | -0.07 (-0.14, -0.01) p=0.033† | 0.04 (-0.04, 0.12) p=0.353† |
| GGT | 16 (13;19) | 13 (11;15) | 14 (13;18) | -0.20 (-0.26, -0.14) p<0.001† | -0.02 (-0.10, 0.05) p=0.55† |
| Basic phosphatase | 275±89.8 | 267±100 | 231.4±101.7 | -9.54 (-23.03, 3.96) p=0.17 | -47.56 (-64.19, -30.94) p<0.001 |
| HbA1c | 34.84±2.5 | 34.56±2.2 | 33.51±1.9 | -0.27 (-0.74, 0.19) p=0.25 | -1.29 (-1.86, -0.72) p<0.001 |
| P-glucose | 5.89±0.4 | 5.85±0.3 | 5.70±0.3 | -0.03 (-0.10, 0.03) p=0.33 | -0.18 (-0.26, -0.09) p<0.001 |
| Albumin | 42.4±2.4 | 42.0±2.4 | 41.5±1.6 | -0.38 (-0.87, 0.11) p=0.13 | -0.80 (-1.40, -0.19) p=0.01 |
| CD-163 | 2.5 (1.9;3.0) | 2.3 (1.8;3.0) | 2.3 (1.9;2.8) | -0.02 (-0.07, 0.03) p=0.44† | -0.09 (-0.16, -0.03) p=0.003† |
| Platelets | 312±59.3 | 276.8±54.6 | 282.5±57.1 | -35.1 (-43.1, -27,2) p<0.001 | -20.6 (-30.4, -10.8) p<0.001 |
| Uric acid | 0.32±0.1 | 0.28±0.1 | 0.29±0.1 | -0.03 (-0.04, -0.02) p<0.001 | -0.01 (-0.02, 0.00) p=0.058 |

**Supplementary S3:** Mixed effect models used to investigate differences in change from baseline to 10-weeks (after camp) and 52-weeks. No adjustments.
† Analyses performed on log-transformed data.
Normal Weight (NW) = BMI-SDS ≤1 SD
Overweight (OW) = BMI-SDS >1SD
Obesity (OB) = BMI-SDS >2SD

| Supplementary S4: Intention-to-treat analysis on changes in physical and metabolic health from baseline to 10 weeks and 52 weeks  (control group and intervention group pooled) | | | | | |
| --- | --- | --- | --- | --- | --- |
|  | **Baseline (n:226)** | **10 weeks (n:200)** | **52 weeks (n:135)** | **Changes from baseline to 10 weeks** | **Changes from baseline to 52 weeks** |
| Sex   - Male - Female | 99 (44%)  127 (56%) | 86 (43%)  114 (57%) | 58 (43%)  77 (57%) | p=0.88 | |
| Weight (kg) | 71.9±16.5 | 65.0±14.2 | 72.3±15.2 | -6.31 (-7.04, -5.57) p<0.001 | 1.91 (1.06,2.77) p<0.001 |
| Height (cm) | 1.59±0.1 | 1.60±0.1 | 1.65±0.1 | 0.01 (0.01,0.02) p<0.001 | 0.06 (0.05, 0.06) p<0.001 |
| BMI-SDS (WHO) | 2.49±0.9 | 1.89±0.9 | 1.97±0.9 | -0.57 (-0.63, -0.52) p<0.001 | -0.44 (-0.50, -0.38) p<0.001 |
| Weight class (WHO)   - NV - OW - OB | 14 (6%)  32 (14%)  180 (80%) | 25 (12%)  81 (41%)  94 (47%) | 20 (15%)  43 (32%)  72 (53%) | p<0.01 | |
| Body fat % | 40.2±8.0, n:215 | 34.2±8.4, n:196 | 34.3±9.0, n:134 | -5.83 (-6.51, -5.14) p<0.001 | -5.16 (-5.95, -4.36) p<0.001 |
| Skeletal muscle mass (kg) | 23.0±5.1, n:215 | 23.1±5.2, n:196 | 25.7±5.5, n:134 | 0.09 (-0.17, 0.34) p=0.51 | 2.78 (2.49, 3.08) p<0.001 |
| Systolic BP | 110.1±10.7, n:191 | 106.2±10.9, n:181 | 110.4±11.1, n:134 | -3.73 (-5.22, -2.25) p<0.001 | 0.73 (-0.93, 2.38) p=0.39 |
| Diastolic BP | 71.0±7.6, n:191 | 67.2±7.3, n:181 | 67.7±8.4, n:134 | -3.58 (-4.72, -2.45) p<0.001 | -2.36 (-3.63, -1.10) p<0.001 |
|  | **n:103** | **n:86** | **n:54** |  |  |
| P-Cholesterol | 4.1 (3.7;4.5) | 3.7 (3.2;4) | 3.8 (3.4;4.2) | -0.49 (-0.61, -0.38) p<0.001 | -0.26 (-0.40, -0.12) p<0.001 |
| HDL Cholesterol | 1.2 (1;1.3) | 1.1 (1;1.3) | 1.2 (1;1.3) | -0.05 (-0.09, -0.01) p=0.02 | -0.02 (-0.07, 0.03) p=0.41 |
| LDL Cholesterol | 2.3 (2;2.6), n:101 | 2 (1.7;2.3) | 2.1 (1.7;2.6), n:52 | -0.35 (-0.45, -0.25) p<0.001 | -0.17 (-0.29, -0.05) p=0.01 |
| P-Triglyceride | 1.2 (0.8;1.6) | 1 (0.8;1.2) | 0.9 (0.7;1.2) | -0.17 (-0.27, -0.07) p=0.001† | -0.18 (-0.30, -0.06) p=0.003† |
| ALAT | 21 (17;30) | 19 (16;25) | 19 (16;23) | -0.16 (-0.25, -0.07) p<0.001† | -0.12 (-0.23, -0.02) p=0.02† |
| ASAT | 23 (18;26) | 21 (18;24) | 23 (20;28) | -0.07 (-0.14, 0.00) p=0.06† | 0.06 (-0.02, 0.15) p=0.16† |
| GGT | 16 (13;19) | 13 (10;15) | 14 (12;18) | -0.19 (-0.26, -0.12) p<0.001† | 0.02 (-0.06, 0.10) p=0.61† |
| Basic phosphatase | 278 (205;327) | 256 (186;322) | 228 (174;288) | 10.10 (-22.07, 1.86) p=0.10 | -43.46 (-57.8, -29.11) p<0.001 |
| HbA1c | 35 (33;36) | 34 (33;36) | 33 (32;35) | -0.27 (-0.69, 0.15) p=0.21 | -1.29 (-1.79, -0.78) p<0.001 |
| P-glucose | 5.9 (5.6;6.1) | 5.8 (5.6;6.1) | 5.6 (5.5;5.9) | -0.03 (-0.09, 0.03) p=0.30 | -0.18 (-0.26, -0.11) p<0.001 |
| Albumin | 43 (41;44) | 42 (41;43) | 41 (40;42) | -0.45 (-0.91, 0.02) p=0.06 | -1.07 (-1.62, -0.52) p<0.001 |
| CD-163 | 2.4 (2;3) | 2.3 (1.8;2.9) | 2.2 (1.9;2.8) | -0.02 (-0.07, 0.03) p=0.45† | -0.07 (-0.14, -0.01) p=0.02† |
| Platelets | 308 (268;347) | 272 (235;313) | 281 (235;319) | -33.48 (-40.87, -26.09) p<0.001 | -19.25 (-28.11, -10.39) p<0.001 |
| Uric acid | 0.31 (0.28;0.36) | 0.28 (0.24;0.32) | 0.29 (0.24;0.34) | -0.03 (-0.04, -0.02) p<0.001 | -0.01 (-0.02, 0.00) p=0.13 |

**Supplementary S4:** Mixed effect models used to investigate differences in change from baseline to 10-weeks (after camp) and 52-weeks. No adjustments.
† Analyses performed on log-transformed data.
Normal Weight (NW) = BMI-SDS ≤1 SD
Overweight (OW) = BMI-SDS >1SD
Obesity (OB) = BMI-SDS >2SD
In the ITT analyses, children with normal weight (n:14), children who were affected by the covid19 lockdown (n:34), children who were identified with a disease or diagnosis (n:4), and children who withdrew from the project or never started camp (n:7) were also included.
